# Clinical and biochemical factors to predict biochemical adrenal insufficiency in hospitalized patients with indeterminate cortisol levels: a retrospective study

**DOI:** 10.1186/s12902-020-0508-7

**Published:** 2020-02-19

**Authors:** Worapaka Manosroi, Natapong Kosachunhanan, Pichitchai Atthakomol

**Affiliations:** 1 Division of Endocrinology and Metabolism, Department of Medicine, Faculty of Medicine, Chiang Mai University Hospital, 110 Intrawarorot Road Soi 2, Si Phum, Amphoe Mueang Chiang Mai, Chiang Mai, 50200 Thailand; 2 Department of Orthopaedics, Faculty of Medicine, Chiang Mai University Hospital, Chiang Mai, Thailand

**Keywords:** Adrenal insufficiency, ACTH stimulation test, Predictive factors, Inpatients

## Abstract

**Background:**

Adrenal insufficiency (AI) in hospitalized patients is a fatal condition if left undiagnosed. Most patients may require an adrenocorticotropic hormone (ACTH) stimulation test to facilitate AI diagnosis. We aim to identify simple biochemical and clinical factors and derive a predictive model to help identify hospitalized patients with biochemical AI who have indeterminate 0800 h serum cortisol levels.

**Methods:**

A seven-year retrospective study was performed in a tertiary care medical center. We identified 128 inpatients who had undergone low-dose or high-dose ACTH stimulation tests. The association between biochemical AI and other factors was evaluated using a logistic regression model clustering by ACTH dose. Stepwise regression analysis was used to demonstrate the predictive model. Diagnostic performance was evaluated using ROC analysis.

**Results:**

Of the 128 patients, 28.1% had biochemical AI. The factors associated with biochemical AI were serum random cortisol < 10 μg/dL (OR = 8.69, *p* < 0.001), cholesterol < 150 mg/dL (OR = 2.64, *p* = 0.003), sodium < 140 mmol/L (OR = 1.73, *p* = 0.004)). Among clinical factors, cirrhosis (OR = 9.05, *p* < 0.001), Cushingoid appearance in those with exogenous steroid use (OR = 8.56, *p* < 0.001), and chronic kidney disease (OR = 2.21, *p* < 0.001) were significantly linked to biochemical AI. The AUC-ROC of the final model incorporating all factors was 83%.

**Conclusions:**

These easy-to-perform biochemical tests and easy-to-assess clinical factors could help predict biochemical AI in hospitalized patients with high accuracy. The physician should therefore have a high index of suspicion to perform dynamic tests for AI diagnosis in those who meet the proposed model criteria.

## Background

Adrenal insufficiency (AI) is the lethal condition in which the adrenal cortex cannot produce sufficient glucocorticoids to support the cellular functions and stress response of the human body [1]. Due to its fatal condition, this disease requires an early diagnosis and immediate treatment [2]. Multiple tests have been proposed to diagnose AI [3]. Serum morning cortisol at 8 AM is an easy and commonly performed measure to screen for AI. If serum cortisol at 0800 h is < 3 μg/dL, it strongly suggests AI, while levels of > 15–18 μg/dL may predict normal adrenal function and AI can be ruled out [4, 5]. When the 0800 h cortisol falls into an indeterminate level and AI is strongly suspected, dynamic tests will need to be employed. The adrenocorticotropic hormone (ACTH) stimulation test is a dynamic test that is usually conducted in clinical practice due to its safety, high reliability and accuracy [6]. This procedure requires measurement of serum cortisol at 30 and 60 min after ACTH was administered at either a low dose (1–5 μg) or a high dose (250 μg). If the peak serum cortisol level after 30 or 60 min is < 18–20 μg/dL, the diagnosis of AI is established [1]. However, in some institutions, the ACTH stimulation test cannot be conducted due to the shortage of ACTH resources and/or no specialists, resulting in diagnostic difficulty. Recent studies have proposed novel cut-off levels for serum cortisol that could reduce the number of unnecessary ACTH stimulation tests. Most of these studies were conducted in the outpatient department [7–9]^.^ Currently, there is only one study that has proposed a proper inpatient cut-off level for serum cortisol [10].

The reported incidence of AI in inpatients is approximately 15.2/10^5^ hospitalized people [11]. The diagnosis of AI in inpatients is fairly challenging due to multiple laboratory sources of interference [12]. Normally, 20% of circulating cortisol is bound to albumin, while all the rest is bound to corticosteroid-binding globulin (CBG). Thus, the measurement of serum cortisol could be interfered by any condition that affects CBG or albumin. For instance, in patients with cirrhosis, nephrotic syndrome or severe sepsis, the levels and binding affinity of CBG to cortisol could be altered [12]. Moreover, individuals with low serum cholesterol, most commonly occurring in chronically ill patients, may have impaired cortisol response to ACTH stimulation tests [13]. Therefore, in this study, we prefer to use the term “biochemical adrenal insufficiency” instead of “adrenal insufficiency” because the ACTH stimulation tests with the proposed cut-off levels have not been validated in acutely ill hospitalized patients who have multiple sources of interference with serum cortisol measurement.

A recent study demonstrated multiple clinical factors associated with biochemical AI in hospitalized patients, e.g., chronic HCV infection, HIV infection, prior orthotropic liver transplantation and reported male hypogonadism [14]. Cirrhosis has been reported to be associated with biochemical AI in multiple studies with a prevalence from 25 to 54% [15, 16]. The association between biochemical AI and serum albumin and cholesterol has been studied [14, 17, 18]. However, combined biochemical factors together with clinical factors to predict biochemical AI in hospitalized patients with equivocal serum cortisol levels have not yet been reported.

The aim of this study was 1) to identify the clinical and biochemical factors associated with biochemical AI in hospitalized patients with indeterminate cortisol levels 2) to create the predictive model for biochemical AI by using both clinical and biochemical factors.

## Methods

A 7-year retrospective study was conducted in the inpatient department at the tertiary care medical center, Faculty of Medicine, Chiang Mai University Hospital, Chiang Mai, Thailand. Data were obtained from electronic medical records of all individuals aged over 18 years who had received ACTH stimulation tests in the inpatient department. Either low-dose (LDT) or high-dose (HDT) ACTH stimulation tests were included during the period of January 2010–January 2017. Before proceeding to ACTH stimulation tests, 0800 h cortisol levels were examined in all patients. The indication to perform either LDT or HDT for hospitalized patients in our institution was a reported level of 0800 h cortisol between 3 and 14.9 μg/dL. The exclusion criteria were patients with septic shock, suspected critical illness-related corticosteroid insufficiency (CIRCI), or incomplete ACTH stimulation test results and women who were currently taking oral contraceptives. Patients who were being treated with glucocorticoids or herbal/traditional medicinal substances suspected of adulteration with glucocorticoids were informed to withhold from those substances 24–48 h before the tests. An electrochemiluminescence immunoassay (ECLIA) method using an Elecsys Cortisol platform (Cobas Roche Diagnostics) was employed to measure serum cortisol levels. Intra- and interassay coefficients of variation for serum cortisol were less than 10%. Total cholesterol levels were measured by an enzymatic, colorimetric method using a Cholesterol Gen.2 platform (Cobas Roche Diagnostics).

### ACTH stimulation testing protocol

All ACTH stimulation tests were conducted by well-trained endocrine nurses. The tests were conducted any time between 0900 h and 1600 h. All patients had an intravenous catheter inserted during the procedure. Total serum cortisol level was measured at 0 (random cortisol), 30 and 60 min after intravenous administration of either 1 μg ACTH for LDT or 250 μg ACTH for HDT (Synacthen®, Tetracosin®, or Cortrosyn®). Only LDT was performed during the period of May 2010–March 2014 due to a shortage of ACTH in Thailand. The 1 μg ACTH dose was prepared by hospital pharmacists under sterile conditions. In brief, a 250 μg ampule of ACTH was diluted with normal saline, packed in 1 mL ready-to-use syringes, and stored at 2–8 °C.

### Definitions

Biochemical AI was defined by a peak serum cortisol level of < 18 μg/dL at 30 or 60 min after LDT or HDT [1]. Random cortisol is serum cortisol drawn anytime before ACTH stimulation tests are performed. In Thailand, the reported incidence of herbal or traditional medicine adulteration with glucocorticoids is high [19]. Therefore, we also documented a history of herbal and/or traditional medicine use as one of the indications for testing. History of herbal/traditional medicine use was positive for patients who reported personally ingested herbal and/or traditional medicinal products dispensed by nonconventional medical practitioners within 3 weeks prior to ACTH stimulation tests. Cushingoid appearance included a constellation of symptoms and signs of corticosteroid excess, e.g., moon face, facial plethora, easy bruising, hirsutism, documented in the medical record by a treating physician. Autoimmune diseases were defined as positive in any patients who had been documented any autoimmune disease as the underlying (systemic lupus erythematosus, rheumatoid disease, autoimmune polyendocrine syndrome, Hashimoto’s thyroiditis, etc.). Chronic kidney disease (CKD) was defined as an eGFR calculated by the MDRD formula of less than 60 ml/min/1.73 m^2^. Orthopedic conditions included any joint pain, back pain, osteoarthritis, bursitis or a history of fracture. The study protocol was approved by the local ethics committee. Significant weight loss was described as a loss of 5% of body weight in one month or 10% over a period of six months or longer that had been documented in the medical record [20].

### Statistical analysis

Data were analyzed using the STATA program (Stata Corp., College Station, TX, USA). Categorical variables are expressed as a number or percentage, and continuous variables are expressed as means and SDs. Statistical analysis of categorical variables was done using the Fisher exact test, while continuous variables were compared using the t-test or Mann-Whitney U test, as appropriate. Univariable analysis was performed by dividing the cohort into 2 groups, which were patients with and without biochemical AI. Multivariable logistic regression analysis was conducted with clustering by ACTH dose (1 or 250 μg) to determine the influence of different biochemical factors on the occurrence of biochemical AI, and odds ratio (ORs) are presented. Demographic and biochemical variables were used to adjust the model. A final diagnostic prediction model was performed by stepwise logistic regression analysis. Stepwise regression analysis was performed manually to identify the smallest number of variables as possible with the highest area under the receiver operating characteristic curve (AUC-ROC). The AUC-ROC of the model was calculated to assess the diagnostic performance. A two-tailed *p*-value < 0.05 was considered statistically significant.

## Results

### Baseline characteristics

Of the 128 inpatients (65 males, 63 females) who had undergone ACTH stimulation testing, 28.1% (36/128) had documented biochemical AI. Baseline characteristics are listed in Table 1. The most common indication for the ACTH stimulation test was history of herbal and/or traditional medicine ingestions and were more commonly observed in the biochemical AI group than in the non-AI group (*p* = 0.021). Other demographic variables, indication for testing, inpatient diagnosis and length of hospital stay were similar between groups. As expected, 0800 h and random cortisol were lower in the biochemical AI group (all *p*-values < 0.001). Apart from serum cortisol, other biochemical values were similar between groups.

**Table 1:** Baseline characteristics

Characteristics	Biochemical adrenal insufficiency (*n* = 36)	No biochemical adrenal insufficiency (*n* = 92)	*p*-value
Age (mean ± SD) (years)	59.0 ± 17.0	55.2 ± 19.5	0.294
Male, n (%)	17 (47.2)	48 (52.1)	0.431
Body weight (mean ± SD) (kg)	53.8 ± 11.4	54.9 ± 14.3	0.712
Underlying diseases, n (%)			
-Hypertension	9 (25.0)	21 (22.8)	0.790
-Coronary artery disease	6 (16.7)	10 (10.8)	0.372
-Autoimmune disease	2 (5.5)	9 (9.7)	0.447
-Malignancy	3 (8.3)	13 (14.1)	0.370
-Chronic renal disease	2 (5.6)	6 (6.5)	0.846
-Cirrhosis/liver disease	4 (11.1)	3 (3.3)	0.073
Indication of tests, n (%)			
-History of exogenous steroid or traditional medicine use	16 (44.4)	22 (23.9)	0.021
-Hyponatremia	8 (22.2)	11 (11.9)	0.141
-Fatigue	11 (30.5)	21 (22.8)	0.367
-Hypotension	8 (22.2)	14 (15.2)	0.342
-Weight loss	4 (11.1)	4 (4.3)	0.153
-Hypoglycemia	1 (2.7)	11 (11.9)	0.104
Cushingoid appearance in patients with exogenous steroid and/or traditional medicine use, n (%)	12 (33.3)	6 (6.5)	< 0.001
Ward, n (%)			
-General medicine	29 (80.6)	76 (82.6)	
-Surgery	7 (19.4)	16 (17.4)	0.782
Inpatient diagnosis			
-Sepsis	11 (30.6)	25 (27.2)	
-Non-sepsis	25 (69.4)	67 (72.8)	0.701
Length of hospital stay (mean ± SD) (days)	22.3 ± 24.1	39.8 ± 41.6	0.349
Baseline SBP (mean ± SD) (mmHg)	109.9 ± 17.5	118.2 ± 21.9	0.060
Baseline DBP (mean ± SD) (mmHg)	67.1 ± 10.2	71.2 ± 15.6	0.174
ACTH stimulation test dose, n (%)			
-1 μg	18 (50.0)	39 (42.4)	
-250 μg	18 (50.0)	53 (57.6)	0.432
Serum 0800 h cortisol (mean ± SD) (μg/dl)	7.4 ± 3.2	10.3 ± 3.1	< 0.001
Serum random cortisol (mean ± SD) (μg/dl)	6.2 ± 3.4	11.6 ± 5.7	< 0.001
Serum albumin (mean ± SD) (g/dl)	2.9 ± 0.8	3.1 ± 0.7	0.162
Serum cholesterol (mean ± SD) (mg/dl)	152.9 ± 45.6	146.0 ± 55.1	0.484
Serum sodium (mean ± SD) (mmol/L)	135.0 ± 6.6	136.5 ± 6.2	0.225
Serum potassium (mean ± SD) (mmol/L)	3.9 ± 0.6	3.9 ± 0.5	0.642
Serum creatinine (mean ± SD) (mg/dl)	1.21 ± 1.1	1.23 ± 1.3	0.956
eGFR	86.4 ± 43.7	99.1 ± 62.0	0.287

### Biochemical predictive factors of biochemical adrenal insufficiency in hospitalized patients

**Fig. 1:**
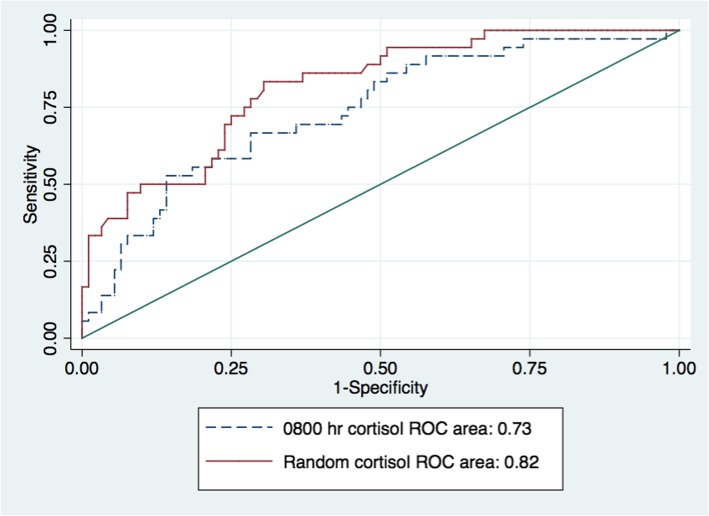
ROC curve of 0800 h cortisol and serum random cortisol for biochemical adrenal insufficiency diagnosis

The diagnostic accuracy of random cortisol in predicting biochemical AI was similar to 0800 h cortisol, as shown in Fig. 1. Serum 0800 h cortisol had a lower AUC-ROC but showed no statistically significant difference comparing to serum random cortisol, with AUCs of 0.73, 95% CI (0.64–0.83) and 0.82, 95% CI (0.74–0.89), respectively, with a *p*-value of 0.16. Due to the sampling of random cortisol is more convenient than 0800 h cortisol, only random cortisol was chosen for analysis in the model.

In the univariate analysis, the only significant biochemical factor predicting the diagnosis of biochemical AI was serum random cortisol. Among clinical factors, history of cirrhosis, Cushingoid appearance in those who used exogenous steroid and/or traditional medicine were significantly associated with biochemical AI. The OR and *p*-value for each variable are shown in Table 2. After multivariate analysis, the significant clinical factors were history of cirrhosis (OR = 9.05, *p* < 0.001), Cushingoid appearance in those who used exogenous steroid and/or traditional medicine (OR = 8.56, *p* < 0.001) and CKD (OR = 2.21, *p* < 0.001). Among biochemical factors, serum random cortisol < 10 μg/dL (OR = 8.69, *p* < 0.001), serum cholesterol < 150 mg/dL (OR = 2.64, *p* = 0.003) and serum sodium < 140 mmol/L (OR = 1.73, *p* = 0.004) were the significant factors linked with biochemical AI.

**Table 2:** Univariate and multivariate odds ratio of biochemical and clinical predictive factors of biochemical adrenal insufficiency in inpatients

Factors	Crude dOR (95% confidence interval)	*p*-value	Multivariable dOR (95% confidence interval)	*p*-value
Cirrhosis	3.70 (1.38–9.91)	0.009	9.05 (7.67–10.66)	< 0.001
Cushingoid appearance in exogenous steroid use	7.16 (3.70–13.87)	< 0.001	8.56 (8.02–9.14)	< 0.001
Chronic kidney disease	0.84 (0.61–1.16)	0.295	2.21 (1.95–2.51)	< 0.001
Random cortisol < 10 μg/dl	8.05 (3.64–17.83)	< 0.001	8.69 (5.76–13.11)	< 0.001
Cholesterol < 150 mg/dL	1.68 (0.77–3.64)	0.189	2.64 (1.37–5.07)	0.003
Sodium < 140 mEq/L	1.53 (1.06–2.19)	0.021	1.73 (1.18–2.53)	0.004

**Fig. 2:**
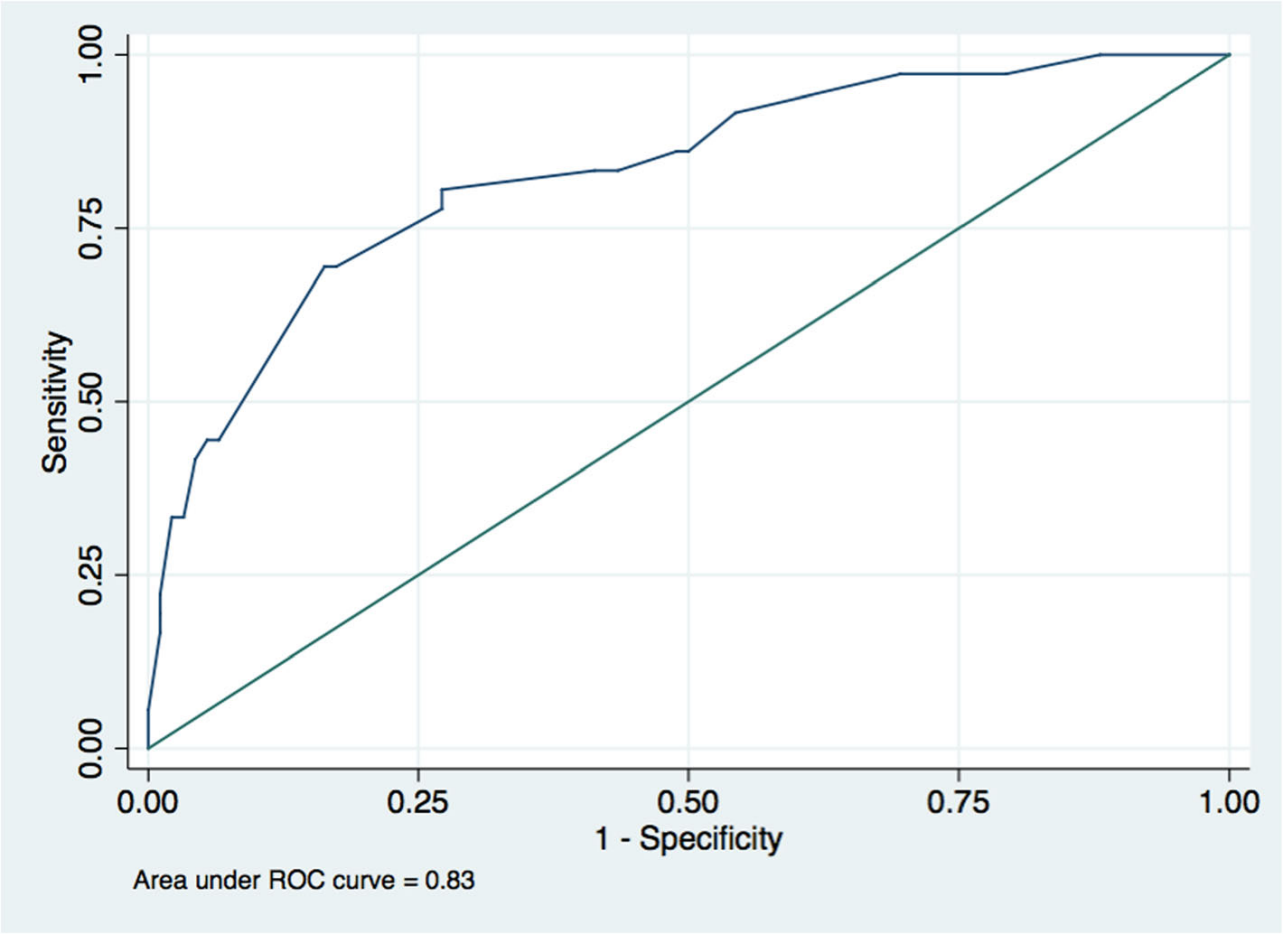
ROC curve of the model incorporating clinical and biochemical factors to predict biochemical adrenal insufficiency

The ROC was plotted to assess the ability of the significant factors to predict biochemical AI in the model, as shown in Fig. 2. An AUC of 83.52% was obtained.

## Discussion

This study highlights an important finding, which is that clinical and biochemical factors can help predict biochemical AI in inpatients. The predictive biochemical factors identified in our study were serum random cortisol, cholesterol and sodium. The clinical factors history of cirrhosis, Cushingoid appearance in those who used exogenous steroid and/or traditional medicine and CKD were significantly predictive. When these factors were combined in the final predictive model, a high diagnostic performance for biochemical AI was demonstrated.

In on our study, 28.1% of the patients who had undergone ACTH stimulation tests had definite biochemical AI. The number is in agreement with a prior study that stated that 23.7% of the patients who underwent ACTH stimulation test were diagnosed with AI [21]. The aforementioned AI incidence in hospitalized patients was lower than our reported number. The explanation for this could be selection bias, as the recruited population in our study was those with highly suspicious AI with indeterminate serum cortisol levels of 3–14.9 μg/dL. These features could have led to the higher incidence of biochemical AI in our population.

Intriguingly, the significant biochemical factors for predicting biochemical AI were serum random cortisol < 10 μg/dL, cholesterol < 150 mg/dL and sodium < 140 mEq/L. All these biochemical factors had ORs of more than 1.0. This could suggest that a higher risk of biochemical AI may occur if there are decreased levels of serum random cortisol, cholesterol and sodium. Serum random cortisol was one of the significant factors linked to biochemical AI. However, the current practice guidelines suggest that there is no evidence to support the use of random cortisol to diagnose AI [1]. We demonstrated that the diagnostic performance shown by the ROC for random cortisol was similar to 0800 h cortisol. Therefore, serum random cortisol was put in the model instead of serum 0800 h cortisol due to its convenience. According to OR, if serum random cortisol is < 10 μg/dL, the risk of biochemical AI increased by 8 times. This result may be applied in routine practice because measuring serum random cortisol in isolation is time-saving, cost-saving and convenient for both healthcare practitioners and patients. Data regarding the higher diagnostic accuracy of random serum cortisol comparing to 0800 h cortisol in diagnosing AI have been described in a previous studies [9, 22].

The association between serum cholesterol and biochemical AI remains controversial. Most studies were primarily conducted in cirrhotic patients. For instance, the study from Park et al. revealed no association between serum cholesterol and AI in cirrhotic patients, while Spadaro et al. stated that cirrhosis with low total cholesterol exhibited an association with AI [17, 23]. In the general hospitalized patient, serum total cholesterol level is not associated with serum random cortisol and AI [14]. In this study, we found that serum cholesterol levels of < 150 mg/dL related to risk of biochemical AI of more than 2 times. In mice treated with simvastatin, AI could be aggravated due to the hypocholesterolemic condition [24]. Since cholesterol is the common precursor for glucocorticoid synthesis, the explanation regarding hypocholesterolemia and biochemical AI could be clarified by testing this hypothesis. In our study, 12.5% of the patients were treated with lipid-lowering medications. However, there was no significant association between biochemical AI and the use of lipid-lowering medications. Likewise, based on the study from patients with cirrhosis, the low level of cholesterol and lipoprotein could influence the adrenal response to ACTH stimulation tests [25]. Therefore, our result regarding low serum cholesterol-linked biochemical AI supports by all these hypotheses.

Serum sodium of < 140 mEq/L was found to be significantly associated with biochemical AI. Our study was comparable to another study that found that in cirrhosis patients, with every 1 mEq/L sodium decrease below 133 mEq/L, there was a significant, 6.41-fold increase in the risk of AI [26]. Likewise, our results demonstrated that if serum sodium was < 140 mEq/L, there was a 1.73-fold higher chance of having biochemical AI. However, a lower risk of AI was observed in our study. This could be explained by the lower incidence of cirrhosis (5%) in our study than in the prior study, in which all patients included were cirrhotic.

Multiple clinical factors related to biochemical AI in hospitalized patients were identified in this study. Each factor demonstrated a very strong association, as each factor had an OR greater than 5, except for CKD. Those with biochemical AI were more likely to have cirrhosis, Cushingoid appearance related to exogenous steroid use and CKD. Ben-Shlomo et al. showed that those with liver diseases were at risk for AI, and our study demonstrated the association between cirrhosis and biochemical AI as well [14]. The link between cirrhosis and biochemical AI may be caused by low cholesterol levels, as discussed earlier. Cushingoid appearance is one of the most common presenting features in those who chronically ingest glucocorticoids [27]. Prolonged intake of corticosteroids with high dosage can result in a negative-feedback inhibition on the hypothalamic-pituitary-adrenal axis (HPA), decreasing the release of ACTH, and AI may occur as a result of reduced corticosteroid synthesis and secretion from the adrenal gland. In our study, CKD was also another significant factor Thus, the risk of biochemical AI may be related to kidney function, but the data related to this issue are still lacking. In patients with CKD, the metabolism and level of serum cortisol may be altered. The plasma cortisol concentration in these patients is elevated or normal, while the normal diurnal variation is preserved [28, 29]. In terms of HPA function, the data are still inconclusive, as one study revealed mild adrenal function impairment in chronic hemodialysis patients, while others showed normal adrenal response [30–32]. These clinical factors could possibly alert healthcare practitioners to perform appropriate dynamic tests early to establish an AI diagnosis. Other factors, including serum potassium and albumin, demonstrated no association with biochemical AI, as in other prior studies [18, 26].

Intriguingly, we found that the best model to predict the occurrence of AI incorporated serum random cortisol, cholesterol, sodium and other clinical factors. Overall, biochemical factors that were integrated into the predictive model can be measured by easy-to-perform laboratory tests, and they are widely available in most hospitals. Moreover, clinical factors in the proposed model are all easy to assess by general practitioners. The accuracy of this model revealed a very high diagnostic performance, with an AUC of 83%. Nevertheless, this model needs to be validated in the future and should be transformed into an easier tool for future application in real clinical practice.

The present study had multiple key strengths. The general biochemical and clinical factors could be employed as predictive factors to diagnose biochemical AI, particularly in those with inconclusive results of serum 0800 h cortisol. Simple laboratory investigations and clinical assessments could be utilized to facilitate AI diagnosis, especially in institutes where ACTH stimulation tests cannot be conducted. The relationships of all the factors could be explained by the underpinning physiology discussed above. All factors that showed significant association were adjusted for confounders by regression analyses. Even though we have included patients who had undergone either low- or high-dose ACTH stimulation tests, statistical analysis by clustering was performed to correct for this variation. Future development of a practical risk score system from the proposed model is required. The number of the studied subjects was adequate and offered enough statistical power (> 90%) for each factor.

We acknowledge some limitations in this study. The population in this study was patients with indeterminate results of serum 0800 h cortisol (3–14.9 μg/dL). Therefore, these results cannot represent the real incidence of AI in the inpatient population. Another limitation is the diverse group of patients; various indications for tests and multiple underlying diseases characterized this cohort. Hence, the results may not be applied to some subsets of the population. However, this limitation itself may have an advantage in terms of the generalization of the results to the general hospitalized population. Also, the results of ACTH stimulation tests after the patients were discharged from the hospital could not be obtained. Therefore, we could not identify whether the patients had persistent biochemical AI or not. As this study is a retrospective study, it could not demonstrate the casual association between the predictive factors and the occurrence of AI. Further prospective research is warranted.

## Conclusion

AI in hospitalized patients is a life-threatening condition that requires early recognition and immediate treatment. ACTH stimulation is still the standard test to diagnose AI. However, there are some areas where the test is unavailable. Although using the proposed simple biochemical and clinical factors could identify some but not all patients with the increased risk of biochemical AI, this proposed model is easy to employ in real clinical practice. At the least, its results could alert the physician to pay close attention to this group of patients and to decide whether they need more rigorous testing for a diagnosis, including further dynamic tests.

## Data Availability

The datasets analysed during the current study are available from the corresponding author on reasonable request.

## Abbreviations

ACTH: Adrenocorticotropic hormone
AI: Adrenal insufficiency
AUC-ROC: Area under the receiver operating characteristic curve
CBG: Corticosteroid-binding globulin
CIRCI: Critical illness-related corticosteroid insufficiency
CKD: Chronic kidney disease
ECLIA: Electrochemiluminescence immunoassay
eGFR: Estimated glomerular filtration rate
HCV: Hepatitis C virus
HDT: High-dose ACTH stimulation test
HIV: Human immunodeficiency virus
LDT: Low-dose ACTH stimulation test
MDRD: Modification of diet in renal disease
OR: Odd ratio
SD: Standard deviation
